# Monitoring Both Extended and Tryptic Forms of Stable Isotope-Labeled Standard Peptides Provides an Internal Quality Control of Proteolytic Digestion in Targeted Mass Spectrometry-Based Assays

**DOI:** 10.1016/j.mcpro.2023.100621

**Published:** 2023-07-20

**Authors:** Rachel A. Lundeen, Jacob J. Kennedy, Oscar D. Murillo, Richard G. Ivey, Lei Zhao, Regine M. Schoenherr, Andrew N. Hoofnagle, Pei Wang, Jeffrey R. Whiteaker, Amanda G. Paulovich

**Affiliations:** 1 Translational Science and Therapeutics Division, Fred Hutchinson Cancer Center, Seattle, Washington, USA; 2 Department of Laboratory Medicine and Pathology, University of Washington, Seattle, Washington, USA; 3 Department of Medicine, University of Washington, Seattle, Washington, USA; 4 Department of Genetics and Genomic Sciences, Mount Sinai Hospital, New York, New York, USA

**Keywords:** targeted mass spectrometry, quantification, immunoaffinity enrichment, clinical proteomics, trypsin digestion, quality control

## Abstract

Targeted mass spectrometry (MS)-based proteomic assays, such as multiplexed multiple reaction monitoring (MRM)-MS assays, enable sensitive and specific quantification of proteotypic peptides as stoichiometric surrogates for proteins. Efforts are underway to expand the use of MRM-MS assays in clinical environments, which requires a reliable strategy to monitor proteolytic digestion efficiency within individual samples. Towards this goal, extended stable isotope-labeled standard (SIS) peptides (hE), which incorporate native proteolytic cleavage sites, can be spiked into protein lysates prior to proteolytic (trypsin) digestion, and release of the tryptic SIS peptide (hT) can be monitored. However, hT measurements alone cannot monitor the extent of digestion and may be confounded by matrix effects specific to individual patient samples; therefore, they are not sufficient to monitor sample-to-sample digestion variability. We hypothesized that measuring *undigested* hE, along with its paired hT, would improve detection of digestion issues compared to only measuring hT. We tested the ratio of the SIS pair measurements, or hE/hT, as a quality control (QC) metric of trypsin digestion for two MRM assays: a direct-MRM (398 targets) and an immuno-MRM (126 targets requiring immunoaffinity peptide enrichment) assay, with extended SIS peptides observable for 54% (216) and 62% (78) of the targets, respectively. We evaluated the quantitative bias for each target in a series of experiments that adversely affected proteolytic digestion (*e.g.*, variable digestion times, pH, and temperature). We identified a subset of SIS pairs (36 for the direct-MRM, 7 for the immuno-MRM assay) for which the hE/hT ratio reliably detected inefficient digestion that resulted in decreased assay sensitivity and unreliable endogenous quantification. The hE/hT ratio was more responsive to a decrease in digestion efficiency than a metric based on hT measurements alone. For clinical-grade MRM-MS assays, this study describes a ready-to-use QC panel and also provides a road map for designing custom QC panels.

## Highlights

• Extended SIS peptides can control for trypsin digestion in targeted MS-based assays.
• Monitoring extended SIS along with tryptic SIS peptides is the basis of a QC metric.
• The ratio of extended to tryptic SIS is a useful in-sample QC metric for digestion.
• The QC metric also correlates with assay sensitivity and quantification fidelity.
• Additional assays can apply this ready-to-use QC panel or identify custom panels.

## Graphical Abstract

**Figure:**
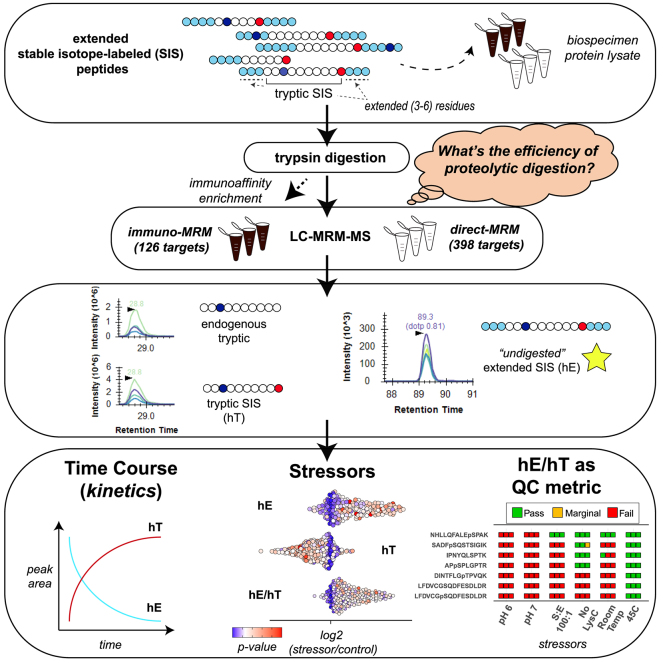

## In Brief

Extended stable isotope-labeled standard (SIS) peptides (hE), proteolytically digested to release the targeted tryptic SIS peptide (hT) form, have been shown to control for trypsin. We found that by monitoring hE along with hT, the resulting hE/hT ratio is a useful in-sample QC metric for digestion efficiency that correlates with endogenous measurement sensitivity and quantification fidelity. This study describes a ready-to-use QC panel as well as a path for identifying custom QC panels that can be applied to additional mass spectrometry-based assays.

Over the past decade, targeted mass spectrometry (MS)-based approaches, like multiplexed multiple reaction monitoring (MRM-MS), have been successfully used to quantify peptides and proteins, including proteins with post-translational modifications (PTMs) (1, 2, 3, 4, 5, 6, 7, 8, 9, 10, 11). Quantitative measurements in MRM-MS assays are typically made using stable isotope dilution in a “bottom-up” fashion, in which proteolytic digestion (*e.g.*, trypsin) converts proteins to peptides, and endogenous proteotypic peptide quantification is performed relative to a spiked, stable isotope-labeled standard (SIS) with the identical peptide sequence (12).

For clinical implementation of targeted MS-based measurements, robust quality control (QC) is a necessary component of the quality management system to ensure reliable diagnostic results (13). In particular, proteolytic digestion efficiency, which is widely regarded as the largest source of variation in quantitative measurements (14, 15, 16, 17, 18, 19, 20, 21, 22, 23), should ideally be monitored *within* individual clinical biospecimens. External QC, performed by analyzing QC materials in parallel with each batch of unknowns, can be useful for monitoring digestion in targeted MS-based proteomic assays, but the complexity of the digestion step and the potential for matrix effects within samples to affect quantitative measurements warrants additional *internal* QC metrics, especially in the clinical setting.

Several approaches using an internal QC for digestion have been developed, including (*i*) monitoring the variation in the intensity of spiked-in tryptic SIS peptides, (*ii*) correlating multiple proteotypic peptides for each protein (24), (*iii*) spiking in an unrelated protein standard (25) to monitor its products, and (*iv*) evaluating the yield of released proteolytic (tryptic) SIS peptides from recombinant SIS protein analogs (22) or extended SIS peptides (26, 27, 28, 29), which recapitulate the tryptic cleavage sites on both the C- and N-termini of the target proteotypic peptide sequence.

While these internal QC approaches can provide some quality assurance, there are some drawbacks to their use. For instance, QC metrics based on tryptic SIS peptides are insufficient to describe the extent of digestion (which can affect assay sensitivity), and their measurements may be confounded by matrix effects specific to individual patient samples. Recombinant SIS proteins have several disadvantages including high cost, light isotopic impurity that adversely affects the limit of detection, and difficulty of synthesis, making them impractical in highly multiplexed assays (16, 30, 31, 32). Furthermore, spiking in additional proteins prior to proteolytic digestion may be undesirable for some samples due to ionization suppression and may not cover all the range of problems with digestion (*i.e.*, it may only be suitable for problems affecting the spiked protein). Thus, even with novel SIS designs, there are no consensus QC approaches for evaluating proteolytic digestion, and effective internal controls monitoring within-sample digestion efficiency across biospecimen samples are needed to apply MRM-MS assays in the clinical environment (33).

To address the need for a robust internal QC of proteolytic digestion in targeted MS-based assays, we hypothesized that MRM-MS measurements of *undigested* extended SIS (hE) peptides, in addition to the tryptic SIS (hT) peptides, could be used as a metric to identify samples in which poor digestion adversely affected endogenous quantification. We tested the ratio of the extended SIS to its paired tryptic SIS peptide, or hE/hT, for its ability to detect variations in proteolytic digestion in two sets of experiments: (*i*) a proteolytic (trypsin) digestion time course experiment and (*ii*) a series of stressor experiments systematically designed to adversely affect proteolytic digestion (*e.g.*, suboptimal pH, temperature, substrate-to-enzyme ratios). We demonstrate the ability of the hE/hT metric to effectively monitor perturbations in proteolytic digestion performance and propose a QC metric based on hE/hT that evaluates proteolytic digestion efficiency and ensures a more sensitive QC metric than one based on the measurements of the tryptic peptide (hT) alone. The proposed QC metric can be implemented in any targeted MS-based proteomics assay using the sequences reported herein. Alternatively, the proposed strategy can be adopted to other assays using the experiments outlined herein to identify those extended SIS sequences most susceptible to changes in proteolytic digestion conditions, and thus SIS pairs best suited to detect poor digestion.

## Experimental Procedures

### Reagents and Standards

Urea (U0631), Trizma (Tris) (pH 8 T2694; pH 7 T1819), citric acid (C0706), EDTA (E7889), iodoacetamide (A3221), and components of a protease inhibitor (PI) mixtures, including PI (P8340), phosphatase inhibitor cocktail 2 (P5726), and phosphatase inhibitor cocktail 3 (P0044), were obtained from Sigma. Tris(2-carboxyethyl)phosphine (TCEP; Bond breaker neutral pH, 77720), Dulbecco’s PBS (14190-144), CHAPS (28300) detergent and LC-MS Optima grade acetonitrile (A955), and water (W6) were obtained from Thermo Fisher Scientific. Other reagents obtained were EGTA solution (pH 8, 40520008) from bioWorld, formic acid (FA, 111670) from Millipore, and Lys-C (129-02541) from Wako. Other proteases used in these experiments were obtained from Promega: trypsin (MS sequencing grade, V5111) and trypsin/Lys-C mix (V5072). A spiked internal retention time (iRT) standard set of 11 peptides was obtained from Biognosys (Ki-3002-1).

Extended SIS peptides were synthesized by Vivitide with sequences corresponding to the tryptic peptide of interest, which contains a fully atom-labeled 13C and 15N on the tryptic C-terminal arginine (R) or lysine (K) residue (if available; otherwise, the incorporation occurred at another amino acid residue: leucine (L), alanine (A), or phenylalanine (F)), along with 3 to 6 amino acids native to the protein on both C- and N-terminal ends of the tryptic sequence (supplemental Tables S1 and S2). For peptide targets of interest that contained PTMs or modified residues, extended SIS peptides were synthesized with the corresponding chemical modifications, including carbamidomethylation of cysteine (+57 Da), phosphorylation of serine or threonine (pS or pT, + 80 Da), or remnant ubiquitination of lysine (uK or K-ε-GG, + 114 Da). All extended SIS peptides were obtained at purity >95% by HPLC, resuspended in 5% acetonitrile with 1% FA, and stored at −80 °C. Molecular mass was confirmed by MALDI-MS, and amino acid stoichiometries and initial concentrations were determined by amino acid analysis (performed by Vivitide). Unlabeled or “light” tryptic peptides corresponding to the proteotypic target peptide were obtained from Vivitide as crude (flash purified) grade for both direct-MRM and immuno-MRM assays for use in method development. Labeled “heavy” tryptic SIS peptides for the immuno-MRM assay were also obtained from Vivitide at > 95% purity. Monoclonal antibodies (supplemental Table S2) were generated and used as previously described (6, 9); many of these are available from the National Cancer Institute’s Antibody Portal (antibodies.cancer.gov).

### Spectral Library Generation of Extended SIS Peptides

A spectral library was generated for undigested, extended SIS peptides to select transitions to target in MRM-MS assays. A mixture of immuno-MRM (126) and direct-MRM (398) extended SIS peptides spiked at 240 fmol/μl (based on the original peptide stock concentrations) into a background of trypsin-digested yeast (*Saccharomyces cerevisiae,* 0.03 μg/μl in 0.1% FA) matrix was analyzed by liquid chromatography (LC) - tandem mass spectrometry (MS/MS) on an Easy-nLC 1000 (Thermo Fisher Scientific) coupled to an Orbitrap Elite mass spectrometer (Thermo Fisher Scientific) or a nanoAcquity (Waters) coupled to an LTQ Orbitrap Velos mass spectrometer (Thermo Fisher Scientific) operated in positive ion mode. For the Elite, the LC system consisted of a fused-silica nanospray needle (PicoTip emitter, 50 μm ID × 20 cm, tip ID 10 ± 1 μm, New Objective, PF360-50-10-N-5) packed in-house with ReproSil-Pur C_18_AQ (3-μm 120 Å resin, Dr Maisch GmbH, r13.aq) with mobile phases of 0.1% FA in water (A) and 0.1% FA in acetonitrile (B). Peptide samples (18 μL) were loaded onto the column and separated over 145 min at a flow rate of 300 nl/min with a gradient from 5 to 7% B for 2 min, 7 to 35% B for 120 min, 35 to 50% B for 10 min, hold 50% B for 1 min, 50 to 90% B for 5 min, hold 90% B for 8 min. A spray voltage of 2750 V was applied to the nanospray tip. MS/MS analysis consisted of one full scan MS from 400 to 1800 m/z at resolution 60,000 followed by 20 data-dependent MS/MS scans using collision-induced dissociation activation with 35% normalized collision energy (CE) of the most abundant ions. Selected ions were dynamically excluded for 15 s after a repeat count of 1. Raw data were converted to mzML and searched using FragPipe (v14.0) against a FASTA protein database consisting of yeast (*S. cerevisiae* UniProt, 6050 proteins, accessed December 2020), extended SIS peptide sequences, and concatenated with a set of randomized sequences. MSFragger (34) search parameters included trypsin enzyme specificity, up to three missed cleavage sites, and variable clipping of N-terminal methionine. Carbamidomethylation of cysteine residues (+57.0215 Da) was set as a fixed modification, and oxidation of methionine residues (+15.9949 Da), phosphorylation of serine, threonine, or tyrosine (+79.9663 Da), and remnant ubiquitination on lysine (+114.0429 Da) were set as variable modifications for those PTMs. In addition, heavy residues on the extended SIS peptides were added as variable modifications, including lysine (+8.014 Da), arginine (+10.008 Da), leucine (+7.017 Da), alanine (+4.007 Da), and phenylalanine (+10.0272 Da). Precursor and fragment mass tolerance was set at 20 ppm. For peptide and protein identification, FragPipe pep.xml files were submitted to PeptideProphet (35) and ProteinProphet, with a false discovery rate set to less than 1% based on the decoy database search. Skyline (36) was used to generate a spectral library for peptides with >90% probability.

### Protein Extraction from GM07057 Mock- and 10Gy-Irradiated Cells

Human lymphoblast cell lines (LCLs) GM07057 were cultured and treated as described previously (9). Briefly, LCLs were either treated with ionizing radiation at 10Gy- or mock-irradiated (handled in the same manner as irradiated cells but the irradiator was not turned on). Cells were harvested one hour post-treatment and pelleted by centrifugation. Pellets were washed twice with ice-cold PBS. Ice-cold urea lysis buffer (6 M urea, 25 mM pH 8 Tris, 1 mM EDTA/EGTA, and 1% PIs) was added directly to cell pellets, and cells were lysed using a microprobe sonicator. Lysates were transferred to microcentrifuge tubes and vortexed before the lysates were centrifuged (20,000*g*, 10 min, 4 °C), and supernatant was collected for further analysis. Lysates were prepared on two separate dates, pooled by treatment, vortexed, bumped down, and aliquoted. Lysate protein concentration was measured by Micro BCA protein assay (Pierce, #23225) following the manufacturer protocol.

### Experimental Design and Statistical Rationale

Samples for LC-MRM-MS analysis were prepared in three independent process replicates (triplicates) to characterize assay variability. Control samples were prepared in both experiments to characterize day-to-day reproducibility.

### Time Course and Stressor Experiments for In-Solution Proteolytic Digestion

Extended SIS peptides were spiked into protein lysates in urea lysis buffer (6 M urea, 25 mM pH 8 Tris, 1 mM EDTA/EGTA, and 1% PIs). For the immuno-MRM assay, 1000 fmol of extended SIS peptide (based on the original peptide stock concentrations) was spiked into 500 μg protein lysate. For the direct-MRM assay, protein lysates were diluted in a higher concentration Tris urea lysis buffer (6 M, 0.5 M pH 8 Tris, 1 mM EDTA/EGTA, and 1% PIs) before 6000 fmol of extended SIS peptide (based on the original peptide stock concentrations) was spiked into 20 μg protein lysate. This higher Tris concentration was to offset any pH changes due to the high percentage of FA after spiking in the 398 direct-MRM panel extended SIS peptides (original peptide stock concentrations prepared in 1% FA). Samples were prepared in process triplicates in Eppendorf protein LoBind deep-well plates (96/2000 μl, 30504305).

**Fig. 1:**
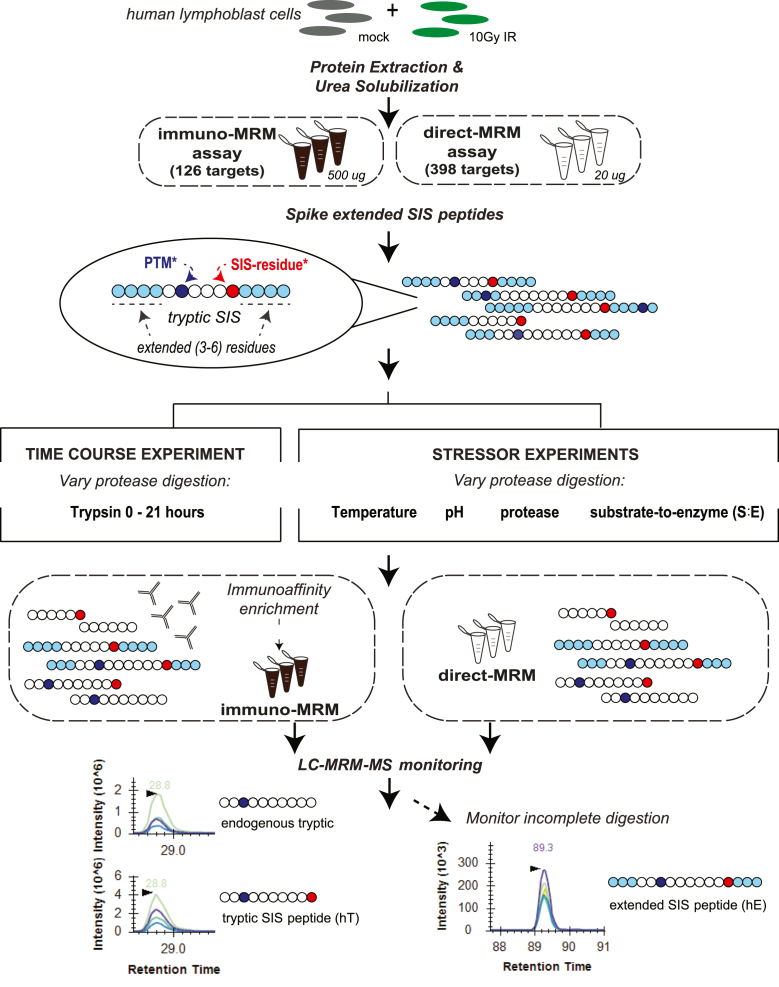
**Experimental design characterizing proteolytic digestion efficiency by measuring extended stable isotope-labeled (SIS), tryptic SIS, and endogenous proteotypic peptides by direct-MRM and immuno-MRM.** Extended SIS peptide standards spiked into protein lysates from human lymphoblast cell lines (LCLs), consisting of mock (untreated) and irradiated (10Gy IR) cells, were analyzed by immuno-MRM (500 μg protein input, 126 peptide targets) and direct-MRM (20 μg protein input, 398 peptide targets) assays. Schematic depicts example extended SIS peptides with amino acid color-coded circles: extended residues beyond the trypsin cleavage sites (*lightblue*), tryptic SIS sequence (*white*), potential post-translationally modified residues (PTM∗, *dark blue*), and fully labeled stable isotope residue (SIS∗, *red*). Along with a control digestion (pH 8 Tris, S/E 50, 37 °C for 16 h), two sets of experiments that affected digestion efficiency were conducted in triplicate: a trypsin digestion time course from 0 min to 21 h (across 10 time points) and digestion stressors, which varied temperature (room temperature or 45 °C), pH of Tris buffer (pH 6 or pH 7), protease used (no addition of Lys-C or a trypsin/Lys-C mix), or substrate-to-enzyme ratio used (S/E 25:1 or 100:1). After the digestion was quenched and desalted, the immuno-MRM targets were immunoaffinity enriched, and targets from both assays were analyzed separately by LC-MRM-MS. LC-MRM-MS analysis included conventional measurements of endogenous tryptic “light” and tryptic “heavy” SIS (hT) peptides as well as novel measurements of *undigested*, extended SIS (hE) peptides. LC, liquid chromatography; MRM, multiple reaction monitoring; MS, mass spectrometry.

Below we describe (*a*) the control digestion sample preparation, then (*b*) how stressor conditions deviated from this control digestion, and finally how the trypsin protease time course experiment was performed (Fig. 1).

(*a*) Control digestion sample preparation: Disulfide bonds were reduced with TCEP (to a final concentration of 30 mM) for 30 min at 37 °C with shaking, followed by alkylation with iodoacetamide (to a final concentration of 60 mM) in the dark at room temperature for 30 min. All protein extracts were then diluted with 0.2 M Tris (pH 8), to reduce the urea concentration to less than 2 M prior to digestion. Protein extracts were then proteolytically digested with sequencing-grade Lys-C for 2 h at 37 °C followed by digestion with trypsin for 16 h at 37 °C on Thermo Mixer heat block. Both Lys-C and trypsin were added at a substrate-to-enzyme (S/E) ratio of 50 (w/w). Protease reactivity was quenched with the addition of FA to a final concentration of approx. 1% v/v.

(*b*) The following deviations for stressor or time course conditions relative to the control digestion protocol were identical for direct-MRM or immuno-MRM assay sample preparation, unless otherwise indicated. For the pH experiments, the pH was adjusted after adding the extended SIS peptides and prior to TCEP disulfide reduction. For direct-MRM pH experiments, the amount of high concentration Tris urea buffer varied relative to normal urea lysis buffer; this resulted in lower Tris-buffering capacity for the high % FA introduced from extended SIS resuspensions and thus, lower pH for conditions pH 6 and pH 7. For immuno-MRM pH experiments, the pH was adjusted by the addition of 1% FA. In addition, for the pH experiments, the dilution of <2M urea step prior to digestion was done using either 0.2 M Tris pH 6 or pH 7. Tris buffer at pH 6 was prepared by the addition of 10% w/v HCl. In all samples, the pH was confirmed at pH 6 or pH 7 prior to and after digestion. For varying the S/E ratio, both Lys-C and trypsin were adjusted to S/E 25 or S/E 100. For varying the digestion temperature, the digestion of both Lys-C and trypsin was in the dark on Thermo Mixer block at room temperature (approx. 20 °C) or at 45 °C. For the no Lys-C experiments, 0.2 M pH 8 Tris was spiked instead for 2 h. For the trypsin/Lys-C mix experiments, Promega sequencing-grade trypsin/Lys-C mix was added instead of Lys-C and incubated 2 h, followed by the addition of 0.2 M pH 8 Tris at the time when trypsin was usually spiked. Lastly, for the trypsin time course conditions, it is noteworthy that protein extracts were first proteolytically digested with sequencing-grade Lys-C for 2 h at 37 °C prior to the start of the trypsin time course. Across 10 time points of the 21-h time course, one sample was sacrificed at each time point and FA was added immediately to quench protease reactivity at 0, 0.083, 0.25, 0.5, 1, 2, 5, 10, 16, and 21 h.

Following FA quench of proteolytic digestion, samples were desalted in the same manner using a positive pressure manifold using an Oasis HLB 96-well plate (30 μm, 10 mg, Waters, 186000128) for the immuno-MRM assay and an Oasis HLB μElution 96-well plate (30 μm, 2 mg, Waters, 186001828BA) for the direct-MRM assay. Oasis plates were first conditioned with 50:50 acetonitrile:water with 0.1% FA and then equilibrated with 0.1% FA in water prior to loading samples. Digested samples were loaded into the Oasis wells and washed with 0.1% FA in water. Peptides were eluted from Oasis wells (<6 psi) using 50:50 acetonitrile:water with 0.1% FA, lyophilized, and stored at −80 °C until immunoaffinity enrichment (immuno-MRM) or resuspension for analysis by LC-MRM-MS (direct-MRM). Prior to injection and analysis by LC-MRM-MS, iRT retention time standard (Biognosys) was spiked at a ratio of 1:10 iRT to sample.

### Peptide Immunoaffinity Enrichment for Immuno-MRM Assay

Enrichment was performed as previously described (6, 9, 37). Monoclonal antibodies for the immuno-MRM panel (supplemental Table S2) were first crosslinked on Protein G Mag Sepharose beads (Cytiva, 28-9513-79), and peptide enrichment was performed using 1 μg antibody-protein G magnetic beads for each target. Briefly, desalted lysate was resuspended in 200 μl 1X PBS with 0.01% CHAPS, transferred to a Kingfisher incubation plate, and the pH was adjusted to 8.0 with 5 μl of 1 M pH 8 Tris. Crosslinked antibodies on beads were mixed well, dispersed, and then immediately spiked into the lysate resuspension. Beads and lysate were incubated >20 h at 4 °C under constant shaking. Following incubation and using a Kingfisher Flex (Thermo Fisher Scientific), beads were washed twice in 1X PBS buffer with 0.01% CHAPS, washed once in 1/10X PBS with 0.01% CHAPS, and peptides were eluted from antibodies in 25 μl of 50 mM citrate containing 5% acetic acid and 3% acetonitrile on a LoBind 96-well PCR plate. The elution plate was covered with adhesive foil and frozen at −80 °C until analysis. Prior to injection and analysis by LC-MRM-MS, iRT retention time standard (Biognosys) was spiked at a ratio of 1:10 iRT to sample.

### Targeted Peptide Analysis by LC-MRM-MS

(*i*) Direct-MRM peptide assay on 6500+ QTRAP. Samples were injected onto the column of an Eksigent Ultra 425 nanoLC system equipped with a chipFLEX system. Sample injection volume was 1 μl. Peptide separation was performed by reversed-phase chromatography using two cHiPLC ReproSil C18 chip columns (each 3 μm, 15 cm × 75 μm; 45 °C) in serial for a total of 30 cm column length. Peptide separation occurred over a 145-min method at a flow rate of 300 nl/min with mobile phase A (0.1% FA in water) and mobile phase B (90% acetonitrile and 0.1% FA in water), starting at 2% B for 0 to 3 min, increasing to 5% B from 3 to 5 min, and then ramping from 5 to 35% B over the course of 120 min. The gradient then switched to 90% at 126 to 131 min followed by a ramp back to starting conditions of 2% B at 132 to 145 min. The Eksigent nanoLC was coupled to QTRAP 6500+ hybrid triple quadrupole/ion trap mass spectrometer (SCIEX) equipped with an OptiFlow source and SteadySpray nano probe (SCIEX). All targeted LC-MRM-MS analyses were carried out in positive ion mode at a spray voltage of 3000 V. The source curtain gas was 20, and interface heating temperature was set to 150 °C. MRM transitions were scheduled with a retention time window of 180 s and a cycle time of 3.0 s. Transitions for each of the 398 direct-MRM analyte tryptic peptides were first confirmed using synthetic light tryptic peptides and using the 398 direct-MRM heavy-extended SIS peptides. Spectral libraries were generated by LC-MS/MS (under the section ‘Spectral library generation of extended SIS peptides’) and imported into Skyline. The top four transitions from those spectral libraries were chosen and CEs were further optimized by LC-MRM-MS. While only heavy transitions were monitored for extended SIS peptides, transitions for both the light tryptic endogenous and heavy tryptic SIS were monitored with a minimum of three transitions chosen for quantification. Of the 398 targets in the direct-MRM assay, 216 (54%) extended SIS peptides were observed by LC-MRM-MS; thus, in total, there were 614 MRM targets (398 tryptic and 216 extended SIS peptides) in the direct-MRM assay (see supplemental Table S3).

(*ii*) Immuno-MRM peptide assay on 5500 QTRAP. Samples were injected onto the column of an Eksigent Ultra 425 nanoLC system equipped with a chipFLEX system. A sample injection of 10 μl was loaded on a trap chip column (Reprosil-Pur C18, 3 μm 0.5 mm x 200 μm, SCIEX) at 5 μl/min for 4 min using mobile phase A (0.1% FA in water). Peptide separation was performed by reversed-phase chromatography using cHiPLC ChromXP C18 chip column (3 μm, 15 cm × 75 μm, 45 °C). Peptides were separated with a flow rate of 300 nl/min, mobile phase A (0.1% FA in water) and mobile phase B (90% acetonitrile and 0.1% FA in water) over a total 50-min method starting at 2% B for 0 to 4 min, increasing to 7% B from 4 to 7 min, ramping from 7 to 25% B for 25 min, and then 25 to 35% B for 5 min. The gradient then switched to 90% at 38 min followed by a ramp back to starting conditions of 2% B at 42 to 50 min. The Eksigent nanoLC was coupled to a 5500 QTRAP hybrid triple quadrupole/ion trap mass spectrometer (SCIEX). The nano electrospray interface was operated in the positive ion MRM mode at a spray voltage of 1300 V. The source curtain gas was 10, and interface heating temperature was set to 110 °C. Parameters for declustering potential and CE were taken from optimized values in Skyline. MRM transitions were scheduled with a retention time window of 180 s and a cycle time of 3 s. Immuno-MRM transition selection and optimization for extended SIS peptides were conducted as described above in (*i*) direct-MRM. Immuno-MRM assays for tryptic peptides have been previously described (6, 9). Of the 126 targets in the immuno-MRM assay, 78 (62%) extended SIS peptides were observed by LC-MRM-MS; thus, in total, there were 204 MRM targets (126 tryptic and 78 extended SIS peptides) in the immuno-MRM assay (see supplemental Table S4).

For both assays, scheduling of MRM transitions was facilitated using Skyline (36) retention time predictor from MRM transitions of Biognosys iRT peptides (38) that were added to the assays (supplemental Table S5).

### Immunoaffinity Enrichment of Extended Heavy Peptides

Anti-peptide antibodies were designed and developed to enrich for tryptic peptides in the immuno-MRM panel (supplemental Table S2). We performed a capture experiment with extended SIS peptides to determine those antibodies that would also capture extended SIS peptides. First, a serial dilution of extended SIS peptides (15.625, 62.5, 250, and 1000 fmol, based on the original peptide stock concentrations) was made in a background of yeast lysate (0.03 μg/μl in PBS) along with a spike of 250 fmol of heavy tryptic SIS peptides (based on the original peptide stock concentrations) and resuspended to 200 μl in 1X PBS, 0.01% CHAPS. All samples were prepared in triplicate. Following the enrichment procedure described above in the ‘Peptide immunoaffinity enrichment for immuno-MRM assay’ section, extended SIS and tryptic SIS peptides in background yeast lysate were captured with anti-peptide antibodies, incubated, washed, and subsequently eluted into 25 μl of 5% acetic acid/3% acetonitrile/50 mM citrate. As a control, another set of serial dilutions of extended SIS peptides with spikes of tryptic SIS peptides were prepared but did not undergo antibody capture. These ‘no-capture’ samples were also prepared in triplicate and resuspended in elution buffer (5% acetic acid/3% acetonitrile/50 mM citrate).

Samples from the extended SIS peptide dilution capture and ‘no-capture’ experiments were immediately analyzed by LC-MRM-MS. Samples were injected onto the column of an Eksigent Ultra 425 nanoLC system equipped with a chipFLEX system. Sample injection volume was 10 μl. The 10 μl injection volumes correspond to 100 fmol of tryptic SIS peptides and a dilution of extended SIS peptides at 6.25, 25, 100, and 400 fmol on-column. Peptides were loaded and separated using the LC method described above in the ‘Targeted peptide analysis by LC-MRM-MS (*ii*) Immuno-MRM peptide assay' section. The Eksigent nanoLC was coupled to QTRAP 6500 hybrid triple quadrupole/ion trap mass spectrometer (SCIEX) equipped with an OptiFlow source and SteadySpray nano probe (SCIEX). All targeted LC-MRM-MS analyses were carried out in positive ion mode at a spray voltage of 2400 V. The source curtain gas was 20, and interface heating temperature was set to 150 °C. Parameters for declustering potential and CE were taken from optimized values in Skyline. Scheduled MRM transitions used a retention time window of 300 s and a desired cycle time of 2.4 s.

### Data Processing and Analysis

LC-MRM-MS data acquired on either the 5500 QTRAP (immuno-MRM assay) or 6500+ QTRAP (direct-MRM assay) were analyzed in Skyline. All peak integrations were reviewed manually, and any transitions with detected interferences were removed from all conditions and not used in the data analysis. An interference was identified by a transition that did not exactly co-elute and/or had a relative intensity deviating more than 20% between the light (or endogenous) and heavy (or SIS) MRM signal. Integrated peak areas were exported and reported as the sum of all transitions. To account for run-to-run fluctuations in overall signal intensity or any variations in ionization efficiency for individual extended SIS (hE) or tryptic SIS (hT) peptide measurements, the raw peak areas were normalized to the peak areas of a set of iRT retention time standard peptides. Herein, “peak areas” will refer to raw peak areas divided by the sum product of 10 of 11 of the iRT peptides (LGGNEQVTR was excluded due to low intensities). Peak area ratios (PARs) of endogenous tryptic “light” peptides to tryptic “heavy” SIS (hT) peptides were used to report target peptide abundances. The lower limit of quantification (LLOQ) of the endogenous “light” peptide peak area was defined as the average light peak area plus 10 times the SD of the light peak area at the digestion condition with the smallest average light peak area. In some cases, there was endogenous signal present in all samples (defined as all samples having a ratio dot product >0.8), so no LLOQ was defined. Hydrophobicity was calculated using SSRCalc (39). Isoelectric point was calculated using EMBOSS (40). Grand average of hydropathy was calculated using GRAVY (41). All physicochemical properties of the extended peptides were calculated with no consideration for modifications.

## Results

We tested the utility of measuring both the released tryptic SIS peptides (hT), as well as the remaining *undigested* extended SIS peptides (hE), for evaluating proteolytic digestion within samples and establishing a QC metric for proteolytic digestion. We used two multiplexed MRM-MS assays (Fig. 1): (*i*) a direct-MRM assay targeting 398 peptides representing 246 proteins related to immune function and (*ii*) a previously described (6, 9) immuno-MRM assay, which couples immunoaffinity enrichment to MRM-MS analysis, targeting 126 unmodified or PTM-containing (phosphorylated or ubiquitinylated) peptides representing 62 proteins related to the DNA damage response network.

### Development of MRM-MS Methods Inclusive of Extended SIS Peptides

To incorporate MRM transitions of the hE peptides into the two MRM-MS methods, synthetic hE peptides (supplemental Tables S1 and S2) representing all targeted proteotypic peptides in these two assays were spiked into a digested yeast matrix and analyzed using data-dependent, shotgun LC-MS/MS on an Orbitrap high-resolution mass spectrometer to generate extended SIS peptide spectral libraries. By LC-MS/MS, 78 of the 126 (62%) hE peptides included in the immuno-MRM assay and 216 of the 398 (54%) hE peptides included in the direct-MRM assay were identified (>90% peptide probability). Extended SIS peptides are less likely to be identified than the tryptic SIS peptides, due to their higher charge states and lower mass-to-charge ratios decreasing the probability of a high confidence peptide identification (42). MRM transitions were selected from spectral libraries constructed from the shotgun LC-MS/MS results to monitor the observed hE peptides by LC-MRM-MS. We used the synthetic hE and hT peptides to empirically determine the optimum precursor charge state, select the top ≥3 MRM transitions, and optimize CEs (the immuno-MRM assay hT peptides were previously optimized (6, 9)). The final direct-MRM method consisted of 1012 precursors and 3775 transitions targeting 614 peptides (398 tryptic and 216 extended SIS) from 246 proteins (supplemental Table S3). The final immuno-MRM method consisted of 330 precursors and 1310 transitions targeting 204 peptides (126 tryptic and 78 extended SIS observable by shotgun LC-MS/MS) from 62 proteins (supplemental Table S4). Finally, each MRM-MS method also included transitions for 11 iRT (Biognosys) retention time standard peptides (supplemental Table S5) that were spiked-in just prior to LC-MRM-MS analysis.

Since tryptic peptides were used as immunogens during monoclonal antibody generation for the immuno-MRM assay (6, 9), we next asked if these antibodies could also capture extended SIS peptides. Triplicate serial dilutions of hE peptides along with a constant amount of hT peptides were spiked into digested yeast background matrix and captured with anti-peptide antibodies (supplemental Tables S2 and S6); controls did not undergo antibody capture. The dilution series was analyzed by LC-MRM-MS, and hE peptides were considered to be effectively captured if at least the top three concentration points in the dilution series had a linear regression fit with R^2^ > 0.97 and slope within 10% of expected (based on the fold dilution). Of the 78 hE peptides observable by shotgun LC-MS/MS (see above), 37 (47%) hE peptides met these criteria. For those hE peptides that were not captured, it is likely that C- or N-terminal extension of the amino acid sequence obscured the antibody epitope.

### Trypsin Digestion Time Course Experiments Demonstrate That the Time Required for hT, Tryptic Light, and PAR Measurements to Reach a Steady State is Peptide-Specific

The multiplexed MRM-MS assays were first applied to a trypsin digestion time course experiment, and the hydrolysis and disappearance of hE peptides along with the production of hT and endogenous “light” tryptic peptides were monitored (Fig. 1). As shown in Figure 1, all extended SIS peptides were spiked into a pooled protein lysate consisting of a mixture of human LCL cells treated with ionizing radiation or mock-irradiated; this mixture was chosen to maximize the representation of endogenous target proteins observable across both the immuno-MRM (6, 9) and direct-MRM assays. Ten time points, ranging from 0 min to 21 h, were collected from the trypsin digestion time course, with the control digestion defined as the 16-h time point. For the immuno-MRM assay, immunoaffinity enrichment was performed prior to LC-MRM-MS analysis. Data for the trypsin digestion time course experiment are available in supplemental Tables S7 and S8.

**Fig. 2:**
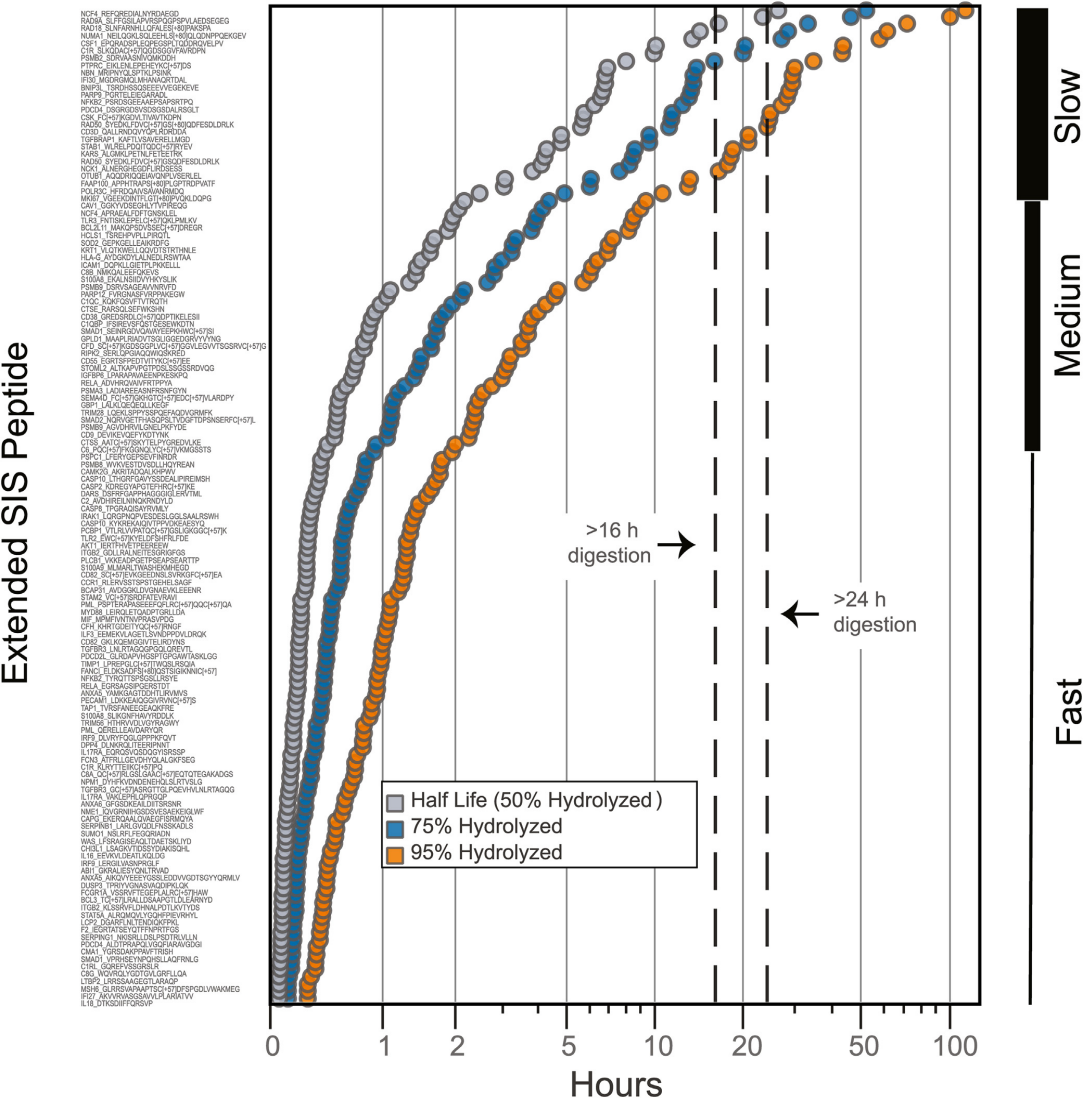
**Hydrolysis half-lives of extended SIS peptides calculated using apparent first order kinetic rate constants.** Plot displaying individual extended SIS peptide hydrolysis half-lives or 50% hydrolyzed extended SIS peptides, as well as calculated times for extended SIS peptides to reach 75% or 95% hydrolyzed for direct-MRM and immuno-MRM targets. Hydrolysis or disappearance of extended SIS peptides across both assays were categorized as fast, medium, slow, or pre-digested by Lys-C (not included in plot) by rate constants calculated using apparent first order kinetics. Time (hours) displayed in logarithmic scale. Dotted references lines indicate conventional overnight proteolytic digestion times of >16 h or >24 h, with the 16-h time point defined as the control digestion. MRM, multiple reaction monitoring; SIS, stable isotope-labeled standard.

Because trypsin hydrolysis follows apparent first order degradation kinetics (43), a first order observed rate constant, *k*_*obs*_, was calculated for the hydrolysis of individual hE peptides. By plotting the natural log as a function of time, or ln(A_t_/A_0_)∼t, with A_t_ and A_0_ reported as the average peak area of hE from process triplicates at time t and time zero, respectively, a linear fit was calculated and *k*_*obs*_ was reported as the slope of the fit (supplemental Table S9). Of the 241 observable hE peptides from both direct-MRM and immuno-MRM assays, 137 had a linear fit with an R^2^ > 0.8 over at least three time points. The other 115 hE peptides were not detectable in the time course experiment, possibly due to being ‘pre-digested’ by Lys-C (added 2 hours prior to the trypsin digestion time course) or ion suppression from the background matrix. The hydrolysis half-life (or 50% hydrolyzed extended SIS peptide), as well as the time needed for hE peptides to reach 75% and 95% hydrolyzed, was calculated from the *k*_*obs*_ (Fig. 2). In the immuno-MRM assay, there was no significant difference in the half-lives of phosphorylated and nonphosphorylated targets. Based on the time needed to reach 95% hydrolysis, the hE peptides were categorized into three groups (Fig. 2): fast digestors (reaching 95% hydrolysis between 0 h–2 h, n = 75), medium digestors (2 h–10 h, n = 34), and slow digestors (>10 h, n = 26).

Measurements from the trypsin digestion time course experiment demonstrated that, as expected, as digestion time increased, the median peak areas of hT and tryptic light increased (supplemental Fig. S1, *A* and *B*), while the median peak areas of hE decreased (supplemental Fig. S1*C*). Furthermore, it appears the production of hT and tryptic light reach a steady state around 10 h, but the PAR of tryptic light to heavy levels off much earlier (supplemental Fig. S1*D*). These data suggest that production of the tryptic light and heavy peptides occurs at approximately the same rate. Note at very short digestion times (less than 2 h), the PAR does not reflect an accurate measure of the amount of light peptide for a subset of the peptides, due to faster release of the SIS peptide than the light endogenous peptide.

### Digestion Stressor Experiments Demonstrate that the hE/hT Ratio is More Sensitive than Either hE or hT to Digestion Perturbations

We next assessed how perturbations to the digestion procedure affected peak areas of hE and hT, as well as the ratio of hE/hT. Stressor experiments were systematically designed to stress or disrupt efficient proteolytic digestion by varying sample preparation conditions, including sample pH, temperature of digestion, substrate-to-enzyme ratio, and removing or adding proteases (Fig. 1). The corresponding results from direct-MRM and immuno-MRM assays were then compared to the control condition, a 16-h trypsin digestion (pH 8, S/E 50:1, 37 °C) (data are available in supplemental Tables S10 and S11).

**Fig. 3:**
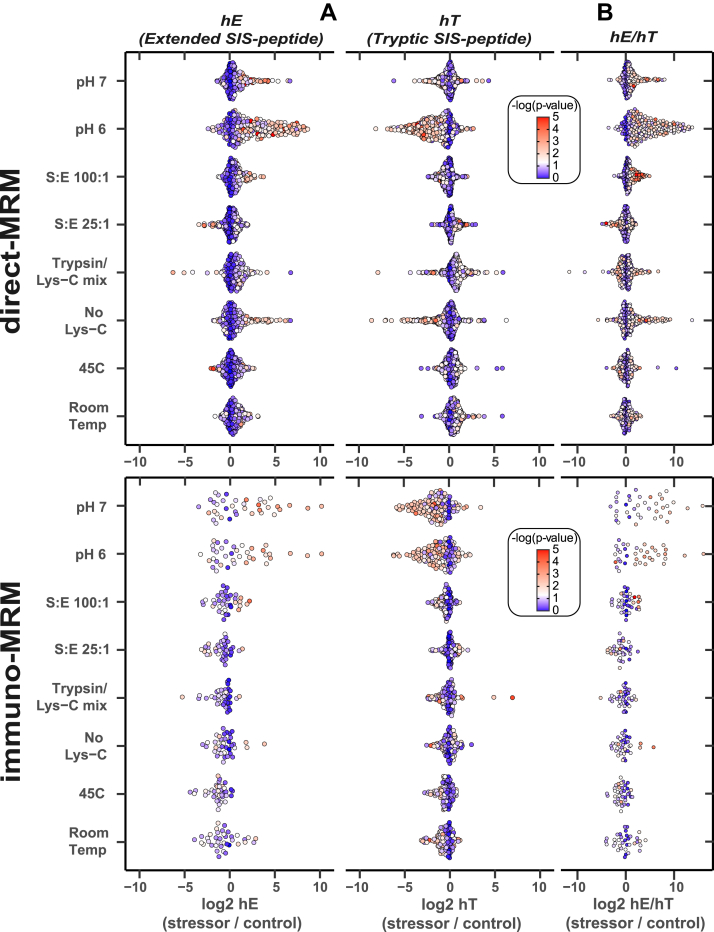
**Swarm-volcano plots fromstressor experiments display significance of peak area fold change compared to control.** The log2 fold change of peak areas from (*A*) individual extended SIS (hE) and tryptic SIS (hT) peptides or (*B*) the ratio of paired extended SIS to tryptic heavy (hE/hT) were calculated from the peak areas averaged across process triplicates in each stressor condition compared to the control digestion (16-h, non-stressed digestion condition). For the direct-MRM assay, 398 heavy tryptic and 216 extended SIS peptides were included in the analysis, and for the immuno-MRM assay, 126 heavy tryptic and 37 extended SIS peptides were included. Circles depict individual peptides in (*A*) and SIS pairs in (*B*). Circle color represents the negative log of the *p*-value, ranging from *blue* (non-significant) to *red* (significant), with white reflecting a *p* value of 0.05. MRM, multiple reaction monitoring; SIS, stable isotope-labeled standard.

We hypothesized that stressing the digestion conditions would affect the hydrolysis of individual extended SIS peptides. Swarm-volcano plots in Figure 3*A* display the log2 fold change of individual hE or hT for each stressor condition compared to the control, with the significance of the changes highlighted by marginal *p*-values (calculated using two-tailed Student’s t-test). Significant differences in hT or hE (highlighted in red, *p*-value <0.05) were most prevalent in the pH perturbation studies for both MRM-MS assays, with many hT significantly lower and hE significantly higher in the stressed digestion than the control (Fig. 3*A*). As expected, plots pairing extended SIS to its released tryptic SIS peptides further illustrated that significantly lower hT corresponded to significantly higher hE within SIS pairs (supplemental Fig. S2). While it is apparent that changes to the pH resulted in some of the most inefficient conditions for hE peptide proteolysis, several other conditions, such as lack of the additional protease Lys-C (no Lys-C; only trypsin added) and increasing the substrate-to-enzyme ratio of trypsin (S/E 100), also reduced the efficiency of extended SIS digestion across both MRM-MS assays (Fig. 3*A*). Interestingly, both the trypsin/Lys-C Promega mix and S/E 25 conditions improved digestion conditions for some target peptides, demonstrated by significantly lower hE and significantly higher hT than control conditions.

Swarm-volcano plots displaying the ratio of hE/hT for SIS pairs (Fig. 3*B*) demonstrated that the magnitude and significance of the hE/hT fold change was higher than the magnitude and significance of the hE or hT peak areas, individually, in each stressor condition compared to control. Higher hE/hT were associated with conditions that had poor extended SIS digestion (*e.g.*, pH 6), and lower hE/hT were associated with conditions more similar to the control digestion (*e.g.*, 45 °C). Analogous plots (supplemental Fig. S3) show the effects of less-than-optimal digestion time, in which significantly higher hE/hT fold changes were observed at shorter digestion times than the control, 16-h digestion. Since the hE/hT ratio was more sensitive than either hE or hT to digestion perturbations across our two experimental settings, we further tested the use of hE/hT as a potential QC metric of proteolytic digestion in multiplexed MRM-MS assays, as described below.

### The hE/hT Ratio Provides a QC Metric of Proteolytic Digestion in Multiplexed MRM-MS Assays

We next compared the utility of the peak area of hT and the ratio of hE/hT as a QC metric to monitor trypsin digestion efficiency. Measurements from the triplicate control digestion samples were used to define the acceptability criteria for the QC metric. A QC pass was defined as an hT value or hE/hT ratio within 2 SD of the median. A marginal QC was defined as a value that was less than three SD but more than two SD greater than the median. Finally, a QC failure was defined as a value that was three SDs greater than the median. We then tested the ability of the QC metric, as defined by either the hE/hT ratio or hT alone, to detect a loss of assay sensitivity, defined as the endogenous peak area in the stressed condition as a fraction of the control. There were 23 targets (15 for the direct-MRM assay, 8 for the immuno-MRM assay) that had endogenous measurements above the LLOQ in the control digestions and also had extended peptides that were not categorized as ‘pre-digested’. Across all stressor conditions, a QC pass/fail rate was then determined by combining the QC results of individual peptides and conditions for which the resulting assay sensitivity was very poor (0%–25%, sensitivity compared to control), poor (25%–50%, sensitivity), fair (50%–75%), good (75%–100%), or equivalent (>100%). We found a higher instance of QC failures among the hE/hT results, demonstrating that QC metrics based on hE/hT were more responsive to losses in assay sensitivity than QC metrics based on hT alone (supplemental Fig. S4).

Next, we identified a subset of SIS pairs for which the hE/hT ratio reliably and sensitively detected inefficient proteolytic digestion during stressor experiments using the following criteria: (*i*) hE/hT was significantly different (*p*-value <0.05) and at least two-fold greater in at least two stressor conditions compared to control and (*ii*) the extended SIS peptide was not ‘pre-digested’ by Lys-C. Finally, to ensure the hE/hT QC metric was not subject to day-to-day variability, we excluded any SIS pairs that failed QC across our replicate, 16-h control digestions (one in the stressor experiment and one in the time course experiment). From these criteria, a QC panel consisting of 36 SIS pairs was selected for the direct-MRM assay, and 7 SIS pairs were selected for the immuno-MRM assay (supplemental Table S12).

To highlight the effectiveness of monitoring digestion efficiency by these QC panels, we plotted the QC pass/fail status of each SIS pair in the time course and stressor experiments (supplemental Fig. S5). Stressor conditions and digestion time points that were associated with the most perturbed digestions (*i.e.*, those with hE/hT that deviated furthest from the controls) had more instances of QC failure than stressors and time points with less perturbed digestions. For instance, all SIS pairs in the QC panel for both direct-MRM and immuno-MRM failed QC under pH 6 stressed conditions, while at 45 °C, temperature digestion conditions in most of the SIS pairs passed QC. As well, the trypsin digestion time course experiment highlights how the QC panel is sensitive to the completeness of digestion, with an increasing number of SIS pairs passing QC as the digestion time increases. We observed a small number of peptides (*e.g.* YTELPYGR, VLDEATLK) that pass QC, then fail (or marginally pass) the QC at the latest time point. This may be indicative of loss of the heavy tryptic peptide at the longest time point, due to adsorption on tube walls. In addition, there are some peptides (*e.g.* GEEDNSLSVR) with inconsistent QC results across the three replicates prior to the last time point. This may be due to the presence of semi-tryptic heavy peptide, which is not considered in the measurement. Interestingly, ‘slow digesting’ extended SIS peptides represent a higher percentage of the panel (33%) than the overall target peptides (19%). We observed that, in general, slower digesting peptides were more likely to fail QC than faster digesting peptides. Across all peptides in the stressor experiment, those that failed QC more than half of the time have a median hydrolysis half-life more than three times longer than those that passed QC more than half of the time (44 min to 14 min). Other physicochemical properties of the extended SIS peptide (supplemental Table S9) do not seem to be predictive of QC pass/fail, as we found that the hydrophobicity, isoelectric point, and grand average of hydropathy were not significantly different between peptides that fail and those that pass the majority of the time.

**Fig. 4:**
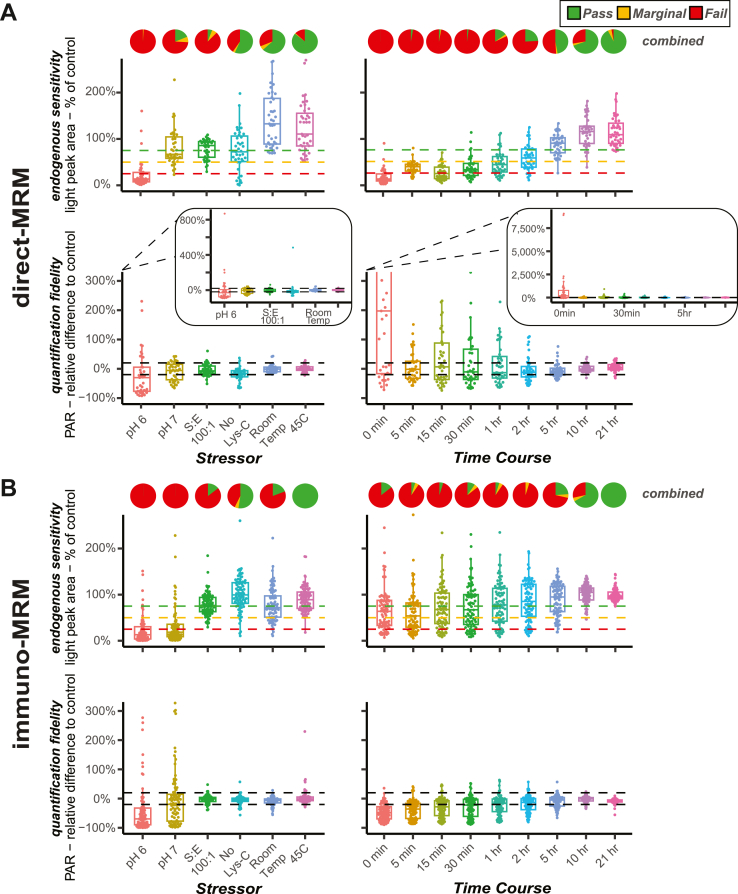
**QC metrics can identify samples with suboptimal endogenous sensitivity and quantification fidelity**. For the (*A*) direct-MRM assay and (*B*) immuno-MRM assay, QC results across the whole QC panel of SIS pairs are displayed as pie charts in the “combined” row, represented as a percentage of pass/fail/marginal QC. Color coding of pie charts depicts failed QC (*red*) as an hE/hT value greater than three SDs from the mean of the control digestion (16-h, non-stressed digestion condition), passed QC (*green*) as an hE/hT less than two SDs from the mean, and marginal QC (*yellow*) as an hE/hT between two and three SDs from the mean. Assay endogenous sensitivity was calculated as the percentage of the endogenous peak area in the inefficient digestion condition compared to the control. Assay quantification fidelity was calculated as the relative percent difference of the light to heavy PAR in the inefficient digestion condition and the control. Endogenous signals are reported for 39 (out of 398) direct-MRM targets and 78 (out of 126) immuno-MRM targets that were measured above LLOQ. Box plots show median (*line*), interquartiles (*box*), and 5 to 95th percentile (*whiskers*). *Horizontal dashed lines* in the assay endogenous sensitivity plots represent 25% (*red*), 50% (*yellow*), and 75% (*green*) of the control, and the *black dashed lines* in the assay quantification fidelity plots represent ± 20% error compared to the control. Axes are consistent within each of the sensitivity and fidelity plots for both assay (*A* and *B*) results except for overlayed fidelity plots in (*A*) showing unconstrained y-axes. LLOQ, lower limit of quantification; MRM, multiple reaction monitoring; PAR, peak area ratio; SIS, stable isotope-labeled standard; QC, quality control.

We next examined how results from the QC panel relate to the sensitivity and fidelity of quantifying endogenous tryptic peptides. Using the time course and stressor experiments, we defined “endogenous sensitivity” as the percentage of endogenous light peak area in the test condition compared to the control and defined “quantification fidelity” as the percent difference of the PAR in the test condition compared to the control. Figure 4 shows the assay endogenous sensitivity and quantification fidelity reported for 117 target peptides (Fig. 4*A* displaying 39 peptides in the direct-MRM assay and Fig. 4*B* displaying 78 peptides in the immuno-MRM assay) with endogenous measurements above the LLOQ in the control samples (data are available in supplemental Table S13). As expected, the experimental conditions that most adversely affected proteolytic digestion (*e.g.*, pH 6 or 5-min time point) had the lowest sensitivity and worst fidelity as demonstrated by the larger deviation from the control (Fig. 4. In addition, as the percentage of QC failures in the panel increases (displayed by the pie charts for each condition), sensitivity and fidelity of endogenous measurements decreased (Fig. 4). Notably, the QC metric was most useful in detecting effects on the sensitivity of the assay compared to the fidelity. This observation was most pronounced in the stressor conditions that produced slightly less tryptic peptide but preserved the expected PAR (*e.g.*, S/E 1:100, no Lys-C; Fig. 4). This is an effect of the tryptic SIS peptide’s being released during digestion along with the tryptic light peptide. Overall, these experiments demonstrate that not only did the QC panel detect problems with extended SIS digestion during sample preparation but also that the extent of failures in the QC panel reflected problems in the fidelity and sensitivity of endogenous proteotypic peptide quantification.

## Discussion

This study provides a proof-of-principle demonstration for a QC approach within individual samples that utilizes measurements of extended SIS peptides to assess the efficiency of proteolytic digestion during sample preparation for MRM-MS proteomic assays. Extended SIS peptides representing the targeted peptides in two multiplexed MRM-MS assays, a direct-MRM and an immuno-MRM assay, were used as internal standards spiked into protein lysates prior to trypsin digestion and subjected to a series of experiments adversely affecting proteolytic digestion. By measuring undigested extended SIS peptides (hE) and tryptic SIS peptides (hT), along with proteotypic endogenous peptides by MRM-MS, we demonstrated that the ratio of SIS pairs, or hE/hT, could effectively monitor perturbations in proteolytic digestion performance and that a QC metric based on the hE/hT ratio more reliably detected loss of digestion efficiency compared to the QC metric based on hT alone.

The use of an internal sample QC metric, such as hE/hT, is important to monitor sample-to-sample variability in proteolytic digestion and to generate reliable and reproducible proteotypic peptide quantification, especially in clinical laboratories implementing multiplexed MRM-MS assays. The QC approach described in this study has the advantage of being incorporated into every sample (as opposed to external QC measures that rely on a batch-level reference sample) without increasing sample complexity by spiking-in additional components (*e.g.*, adding exogenous protein digestion standards). For both direct-MRM and immuno-MRM assays, the QC results identified individual samples with conditions which perturbed the proteolytic digestion (low pH, low temperature, <5 h, etc.) negatively, impacting the quantification of endogenous peptides compared to control. Across the stressor experiments, a higher percentage of failures in the QC panel related to poor assay sensitivity and fidelity when quantifying endogenous measurements. Thus, this QC approach provides within-sample assurance of efficient proteolytic digestion, ensuring reliable and reproducible proteotypic peptide quantification across individual biospecimens, which is essential for implementing MRM-MS assays in the clinical environment.

Monitoring all targets of SIS pairs in an assay panel is a challenge, since not all extended SIS peptides are amenable to LC-MRM-MS (from this study, 54% from the direct-MRM and 62% from the immuno-MRM assays), and not all extended SIS peptides are efficiently captured by the antibodies generated using the fully tryptic peptides as the antigens (47% from the immuno-MRM assay). Using only a subset of SIS pairs in a QC approach is advantageous because it minimizes the number of MRM transitions and thus analysis time needed to target hE peptides within a method. However, a limitation of not monitoring the hE/hT pair for all targets in an assay is that sample-specific matrix effects on any individual target may be missed.

This QC approach should be amenable to assays using stable isotope-labeled full-length recombinant proteins, QPrEST (44) or QconCAT (45) as internal standards, making this QC metric broadly applicable to targeted MS-based proteomics. The ready-to-use QC panel of extended SIS peptides identified in this study can be spiked into other clinical-grade MS-based assays to monitor digestion. Alternatively, this study also provides a road map for designing custom QC panels by identifying those hE/hT pairs from proteotypic target peptides of interest that could serve as QC metrics in other MRM-MS assays. Finally, due to the low-cost of commercially available extended and tryptic SIS peptides as well as the ease of incorporating and automating measurements of both SIS pairs in software analysis packages, there exist few barriers for widespread implementation of this valuable QC approach within targeted MS-based proteomics platforms.

## Data Availability

All raw data, manually integrated peak areas, transition information, and retention times generated from these stressor and time course experiments for the immuno-MRM and direct-MRM peptide target assays are available *via* Panorama Public (46) at https://panoramaweb.org/Extended_peptide_MRM_digestion_QC.url; MRM and MS/MS output files can be found under the "Raw Data" tab. A summary of and link to the dataset is accessible through the ProteomeXchange Consortium (http://proteomecentral.proteomexchange.org) under the dataset identifier PXD038465. Characterization data for available assays are found in the CPTAC Assay Portal (assays.cancer.gov).

## Supplemental data

This article contains supplemental data containing supplemental figures and supplemental tables.

## Conflict of interest

The authors declare no competing interests.
