# Supplementary material for: Monitoring Both Extended and Tryptic Forms of Stable Isotope-Labeled Standard Peptides Provides an Internal Quality Control of Proteolytic Digestion in Targeted Mass Spectrometry-Based Assays
Source: Mol Cell Proteomics. 2023 Jul 20;22(9):100621. doi: 10.1016/j.mcpro.2023.100621 (PMC10458721; doi:10.1016/j.mcpro.2023.100621)
Supplement: Supplemental information [file mmc1.pdf]

Supplemental Data

Monitoring both extended and tryptic forms of stable isotope-labeled standard peptides provides an internal quality control of proteolytic digestion in targeted mass spectrometry-based assays

Rachel A. Lundeen[1±], Jacob J. Kennedy[1±], Oscar D. Murillo[1], Richard G. Ivey[1], Lei Zhao[1], Regine M. Schoenherr[1], Andrew N. Hoofnagle[2,3], Pei Wang[4], Jeffrey R. Whiteaker[1*], and Amanda G. Paulovich[1*]

AUTHOR INFORMATION

[1]Translational Science and Therapeutics Division, Fred Hutchinson Cancer Center, Seattle, WA, USA

[2]Department of Laboratory Medicine and Pathology, University of Washington, Seattle, WA, USA

[3]Department of Medicine, University of Washington, Seattle, WA, USA

[4]Department of Genetics and Genomic Sciences, Mount Sinai Hospital, New York, NY 10065, USA

[±]These authors contributed equally to this paper.

*Corresponding authors: Amanda G. Paulovich (apaulovi@fredhutch.org) and
Jeffrey R. Whiteaker (jwhiteak@fredhutch.org)

Table of Contents

Figure S1. Effects of trypsin digestion time on extended and tryptic peak areas.

Figure S2. Sample preparation stressor effects on the measurements of the heavy extended and tryptic SIS pairs.

Figure S3. Digestion time points that affect heavy tryptic peptide measurements also affect extended SIS peptide measurements.

Figure S4. QC pass/fail rates of QC metrics based on either hE/hT or hT at different assay sensitivity ranges.

Figure S5. Graphical display of hE/hT QC metric pass/fail for time course and stressor experiments from QC panel of SIS pairs in direct-MRM and immuno-MRM assays.

Table S1. Direct-MRM assay panel: sequences for extended heavy modified peptide and heavy tryptic modified peptide.

Table S2. Immuno-MRM assay panel: sequences for extended heavy modified peptide and heavy tryptic modified peptide.

Table S3. MRM parameters for peptides targeted by the direct-MRM panel.

Table S4. MRM parameters for peptides targeted by the immuno-MRM panel.

Table S5. MRM parameters for Biognosis iRT peptides.

Table S6. Immuno-MRM panel antibody capture experiment with 'undigested' extended heavy peptides.

Table S7. Direct-MRM assay results for trypsin digestion time course experiments.

Table S8. Immuno-MRM assay results for trypsin digestion time course experiments.

Table S9. Rate constant calculations for direct-MRM and immuno-MRM assay targets from trypsin digestion time course experiments.

Table S10. Direct-MRM assay results for stressor experiments.

Table S11. Immuno-MRM assay results for stressor experiments.

Table S12. Development of the hE/hT QC metric and panel.

Table S13. Endogenous peptide quantification.

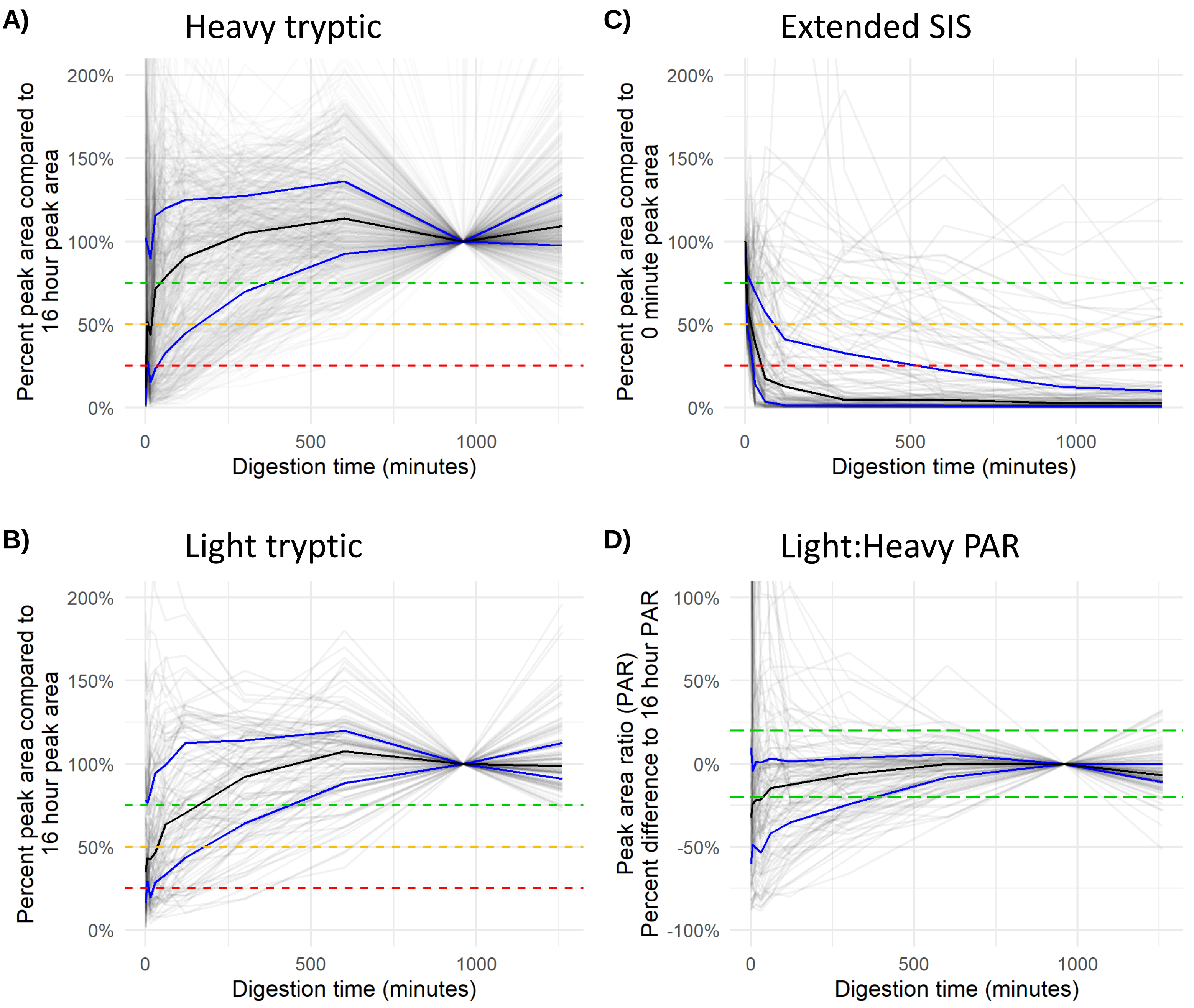

Figure S1: Effects of trypsin digestion time on extended and tryptic peak areas. Compared to the 16 hour (for tryptic peptides) or 0 minute (for extended peptides) time point, the percent peak areas of (a) 524 heavy tryptic, (b) 117 light tryptic peptides and (c) 137 extended SIS peptides, as well as the percent difference of (d) 117 light to heavy tryptic peak area ratios (PAR) for 10 time points spanning 21 hours. Light tryptic peptides with endogenous measurements above the LLOQ in the control samples and heavy SIS peptides that were not 'pre-digested' were included in this analysis. Individual peptides are represented by gray lines, the median value by a black line, and the interquartile range by blue lines. PAR difference of 20% and -20% is represented by dashed green lines.

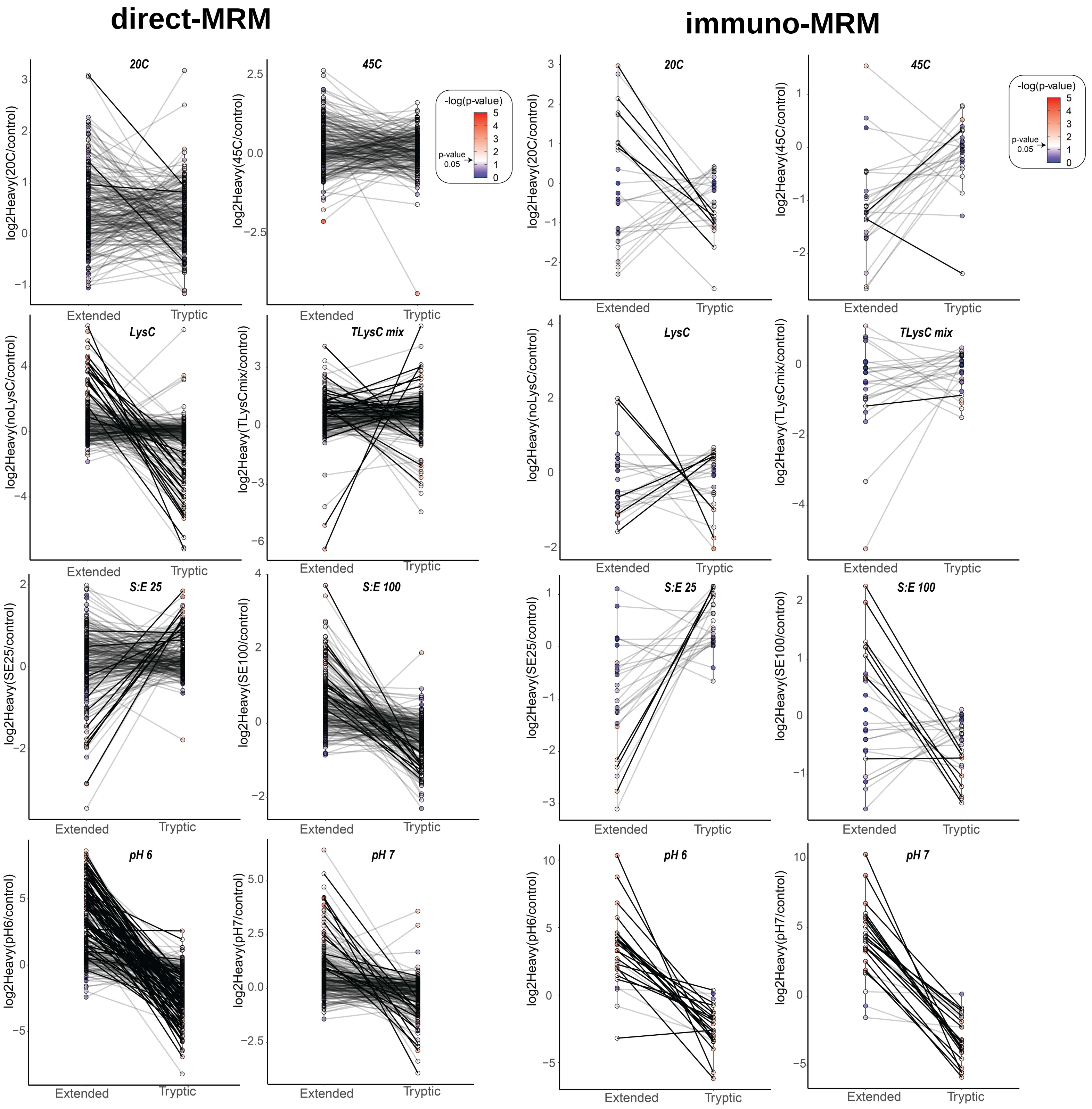

Figure S2: Sample preparation stressor effects on the measurements of the heavy extended and tryptic SIS pairs. For extended and tryptic SIS pairs, the lines connect the peak area fold change compared to control (log2) in the direct-MRM and immuno-MRM assays. Solid black lines represent pairs in which peak areas of both the extended and tryptic SIS peptides are significantly different from the control (red) and gray represent pairs in which peak areas of either one or both of the extended and tryptic SIS peptides are not significantly different from the control (blue).

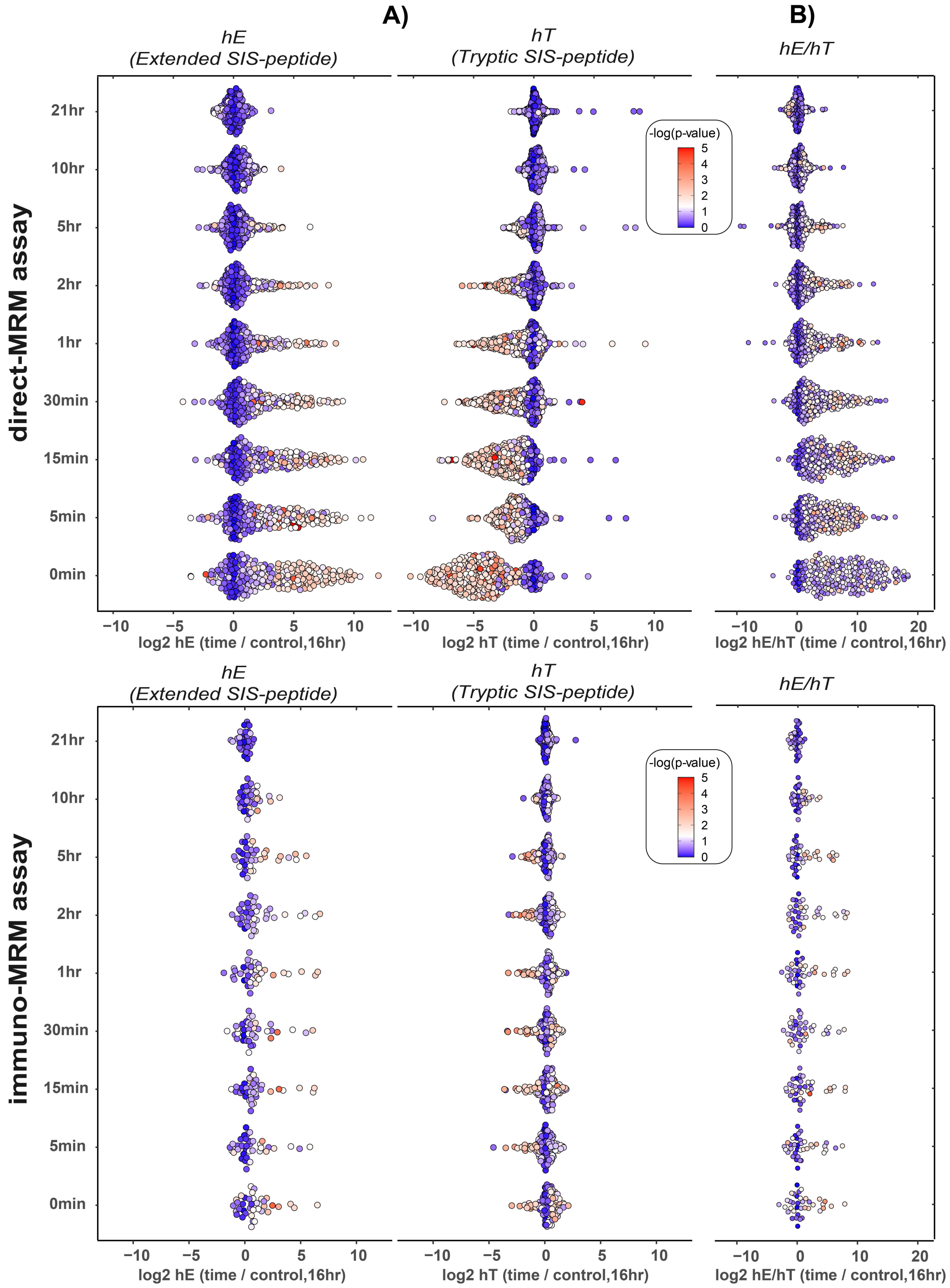

Figure S3: Digestion time points that affect heavy tryptic peptide measurements also affect extended SIS peptide measurements. The log2 fold change of each extended SIS (hE) peptide and tryptic heavy (hT) peptide were calculated for the peak areas in each time point compared to the control digestion (16-hour, non-stressed digestion condition). For the direct-MRM assay, 398 heavy tryptic and 216 extended SIS peptides were included in the analysis and for the immuno-MRM assay, 126 heavy tryptic and 37 extended SIS peptides were included. Each circle represents an individual peptide in (a) hE or hT swarm-volcano plots or peptide pairs in (b) hE/hT swarm-volcano plot. The color-coding for each circle displays the negative log of the p-value, ranging from blue (non-significant) to red (significant); the white transition reflects a p-value of 0.05.

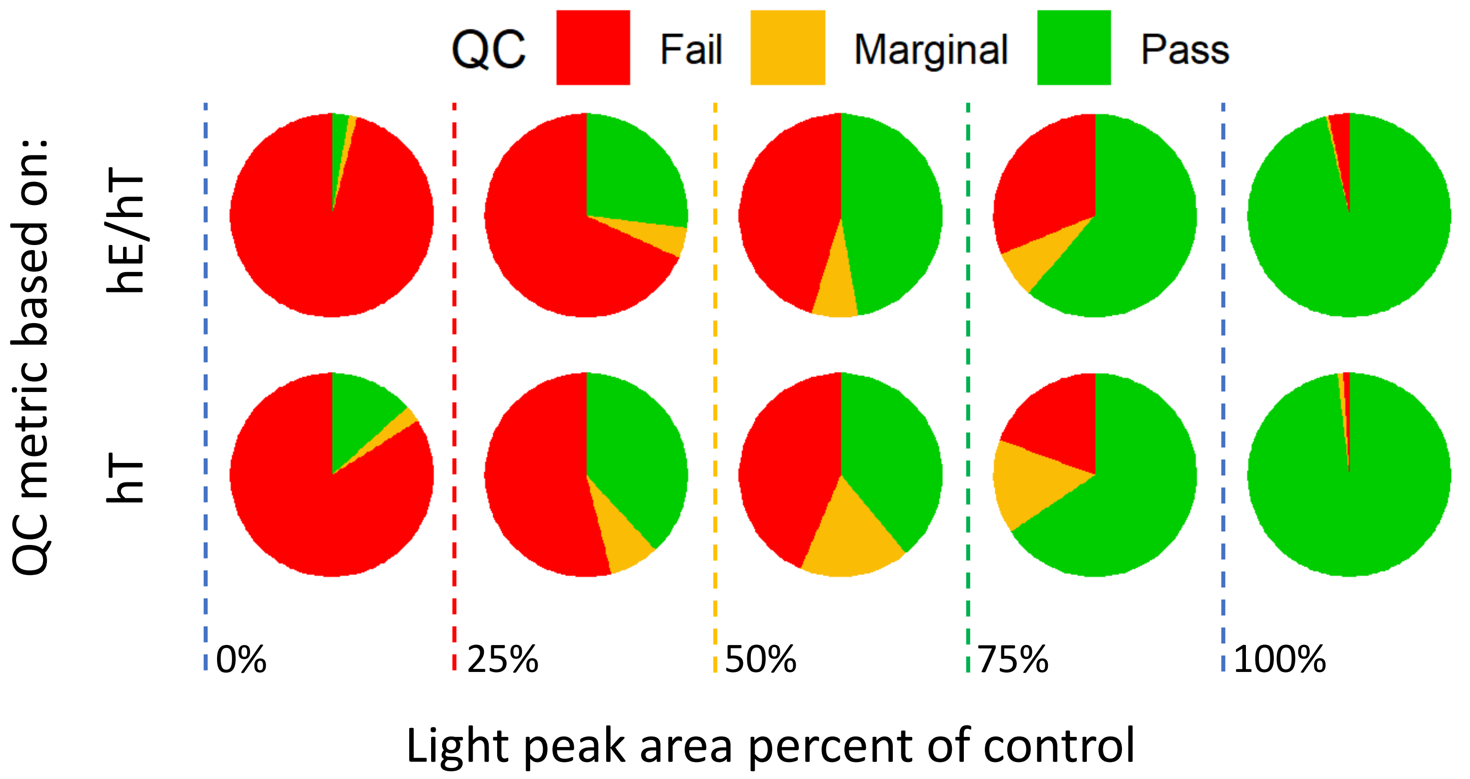

Figure S4: QC pass/fail rates of QC metrics based on either hE/hT or hT at different assay sensitivity ranges. QC metrics were calculated using either hE/hT or hT for 23 targets for which the extended SIS, tryptic SIS and tryptic endogenous versions of the peptide were measurable and not pre-digested. Across all stressor conditions, a QC pass/fail rate was determined by combining the QC results of individual peptides and conditions in which the resulting assay sensitivity was very poor, poor, fair, good or equivalent to the control condition.

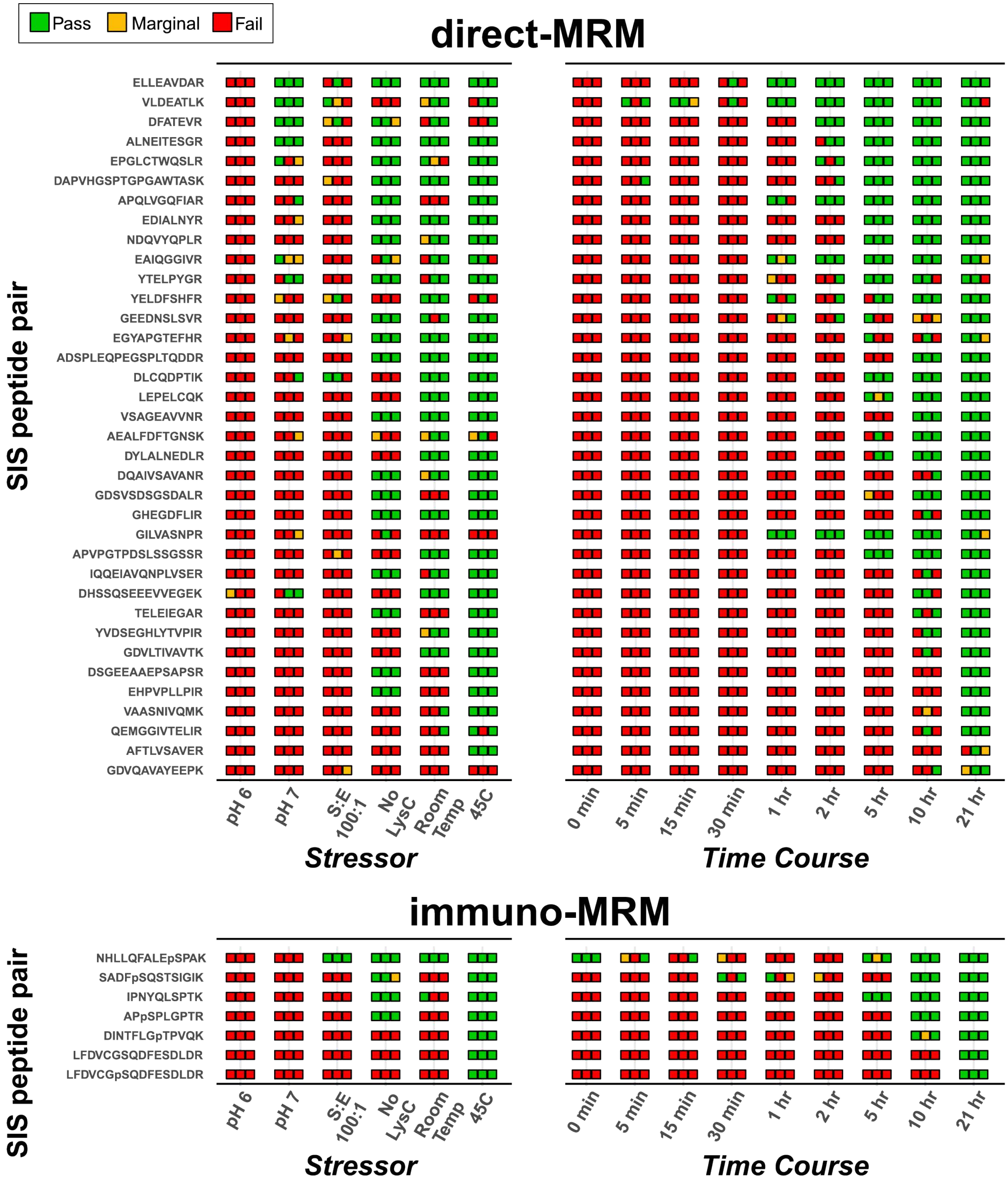

Figure S5: Graphical display of hE/hT QC metric pass/fail for time course and stressor experiments from QC panel of SIS pairs in direct-MRM and immuno-MRM assays. QC results are shown for a QC panel of 36 extended and tryptic SIS pairs in the direct-MRM assay and 7 extended and tryptic SIS pairs in the immuno-MRM assay. Each SIS peptide pair is plotted by tryptic target peptide sequence with each box representing the QC results for one of three replicate samples for each stressor or time-course condition. Color-coding depicts failed QC (red) as an hE/hT value greater than three standard deviations from the mean of the control digestion (16-hour, non-stressed digestion condition), passed QC (green) as an hE/hT less than two standard deviations from the mean, and marginal QC (yellow) as an hE/hT between two and three standard deviations from the mean.
